# Glioblastoma, IDH-wildtype, with spontaneous transdural and calvarial invasion: a case report and literature review

**DOI:** 10.3389/fmed.2026.1908149

**Published:** 2026-10-09

**Authors:** Jianhang Lin, Jiayi Lin, Jiana Zhang, Haiqing Xie, Dawei Liu, Shaolei Guo

**Affiliations:** 1 Department of Neurosurgery, The First Affiliated Hospital, Sun Yat-sen University, Guangzhou, China; 2 Department of Pathology, The First Affiliated Hospital, Sun Yat-sen University, Guangzhou, China; 3 Department of Neurology, The First Affiliated Hospital, Sun Yat-sen University, Guangzhou, China

**Keywords:** calvarial invasion, dural invasion, extracranial extension, glioblastoma, IDH-wildtype

## Abstract

Glioblastoma (GBM), IDH-wildtype, is the most aggressive adult-type diffuse glioma. Although extracranial spread may occur after craniotomy, radiotherapy, or other interventions, spontaneous transdural and calvarial invasion before treatment is exceedingly rare. We report a 37-year-old woman who presented with long-standing right temporal-parietal headache and acute worsening with vomiting. Computed tomography and magnetic resonance imaging demonstrated a right temporal mass with intratumoral hemorrhage, peritumoral edema, and contiguous extension through the dura and temporal bone into the temporalis muscle. The lesion was resected through a transcranial approach, and postoperative imaging showed gross total resection. Histological examination demonstrated an infiltrating high-grade glial neoplasm with brisk mitotic activity and palisading necrosis. Immunohistochemical and molecular findings, including the absence of IDH1/IDH2 mutations, supported a diagnosis of glioblastoma, IDH-wildtype, CNS WHO grade 4. At 18 months after surgery, the patient remained alive. This case highlights a rare pattern of primary extracranial extension in GBM and suggests direct contiguous transdural invasion as a plausible route of spread in the absence of prior intervention.

## Introduction

Gliomas are the most common primary malignant tumors of the central nervous system and are classified according to integrated histological and molecular criteria in the World Health Organization (WHO) classification (1, 2). Glioblastoma (GBM), IDH-wildtype, is characterized by rapid proliferation, diffuse parenchymal infiltration, microvascular proliferation, necrosis, and poor prognosis. Clinical manifestations include headache, seizures, visual disturbance, vomiting, and focal neurological deficits (3). Because GBM infiltrates surrounding brain tissue, complete surgical eradication is rarely achievable, contributing to recurrence and limited survival (4). Extracranial metastasis or extension is reported in fewer than 2% of patients with GBM (5, 6) and most often follows craniotomy, radiotherapy, or other disruption of anatomical barriers (7, 8). Spontaneous invasion of the dura, calvarium, galea, or extracranial soft tissues without prior intervention is therefore exceptional (9–11). Here, we describe a rare case of right temporal GBM, IDH-wildtype, with spontaneous transdural, calvarial, and temporalis muscle invasion in a patient with no previous surgery, radiotherapy, or chemotherapy.

## Case presentation

A 37-year-old woman presented with headache involving the right temporal and parietal regions for more than 2 years, with worsening headache and vomiting during the preceding 9 days. She had a 2-year history of chronic sinusitis but denied a family history of neurocutaneous disorders, childhood tumors, or toxic exposure. Neurological examination revealed no significant deficit. Two round brown pigmented cutaneous lesions, each approximately 1 × 1 cm, were observed on the left middle finger and scalp. Neither lesion underwent dermatological assessment, excision, or histopathological examination; therefore, their nature could not be definitively established. Contrast-enhanced spiral computed tomography (CT) showed a right temporal mass with surrounding vasogenic edema and extension to the dura and right temporal bone. CT perfusion demonstrated relatively increased perfusion within the lesion, supporting a hypervascular high-grade neoplasm but not providing tumor-specific discrimination. Brain magnetic resonance imaging (MRI) demonstrated a large right temporal lesion with intratumoral hemorrhage and associated hydrocephalus. The lesion measured approximately 28 × 41 × 45 mm and showed hyperintensity on both T1- and T2-weighted images. Imaging also demonstrated extension through the skull base with involvement of the right dura, temporal bone, and temporalis muscle (Figure 1). Preoperative positron emission tomography-computed tomography (PET/CT) revealed no evidence of other extracranial lesions or a primary malignancy elsewhere.

**Figure 1:**
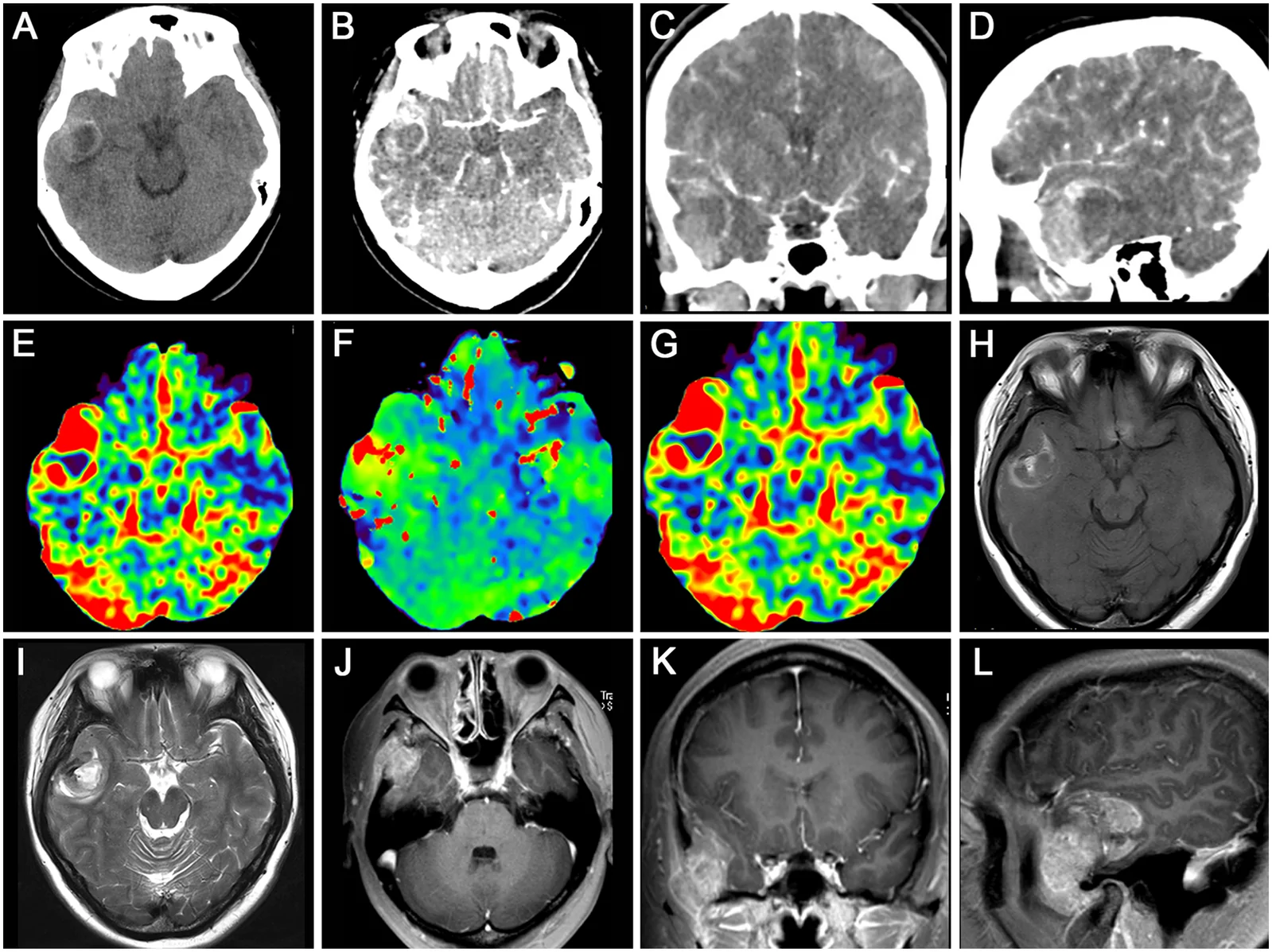
Serial preoperative imaging of the intracranial lesion. **(A–D)** Non-contrast and contrast-enhanced spiral computed tomography (CT) showed a right temporal mass with surrounding vasogenic edema and invasion of the dura and right temporal bone. **(E–G)** Volume-based and flow-based CT perfusion images demonstrated relatively increased perfusion within the lesion. **(H–L)** Brain magnetic resonance imaging (MRI) showed a large right temporal mass with intratumoral hemorrhage and hydrocephalus, with extension through the skull base into the dura, temporal bone, and temporalis muscle.

The patient underwent resection of the intracranial and extracranial communicating tumor in the right middle cranial fossa, together with subtemporal decompression (Figure 2). Postoperative MRI performed within 72 h showed no radiologically evident residual tumor. After resection, the patient received concurrent chemoradiotherapy consisting of 30 fractions of radiotherapy and concomitant temozolomide at 75 mg/m^2^/day for 42 days. She subsequently received adjuvant temozolomide on days 1−5 of each 28-day cycle combined with anti-PD-1 immunotherapy using tislelizumab. Tumor-treating fields (TTFields) therapy was initiated four months after surgery. At 18 months after surgery, the patient remained alive and under follow-up.

**Figure 2:**
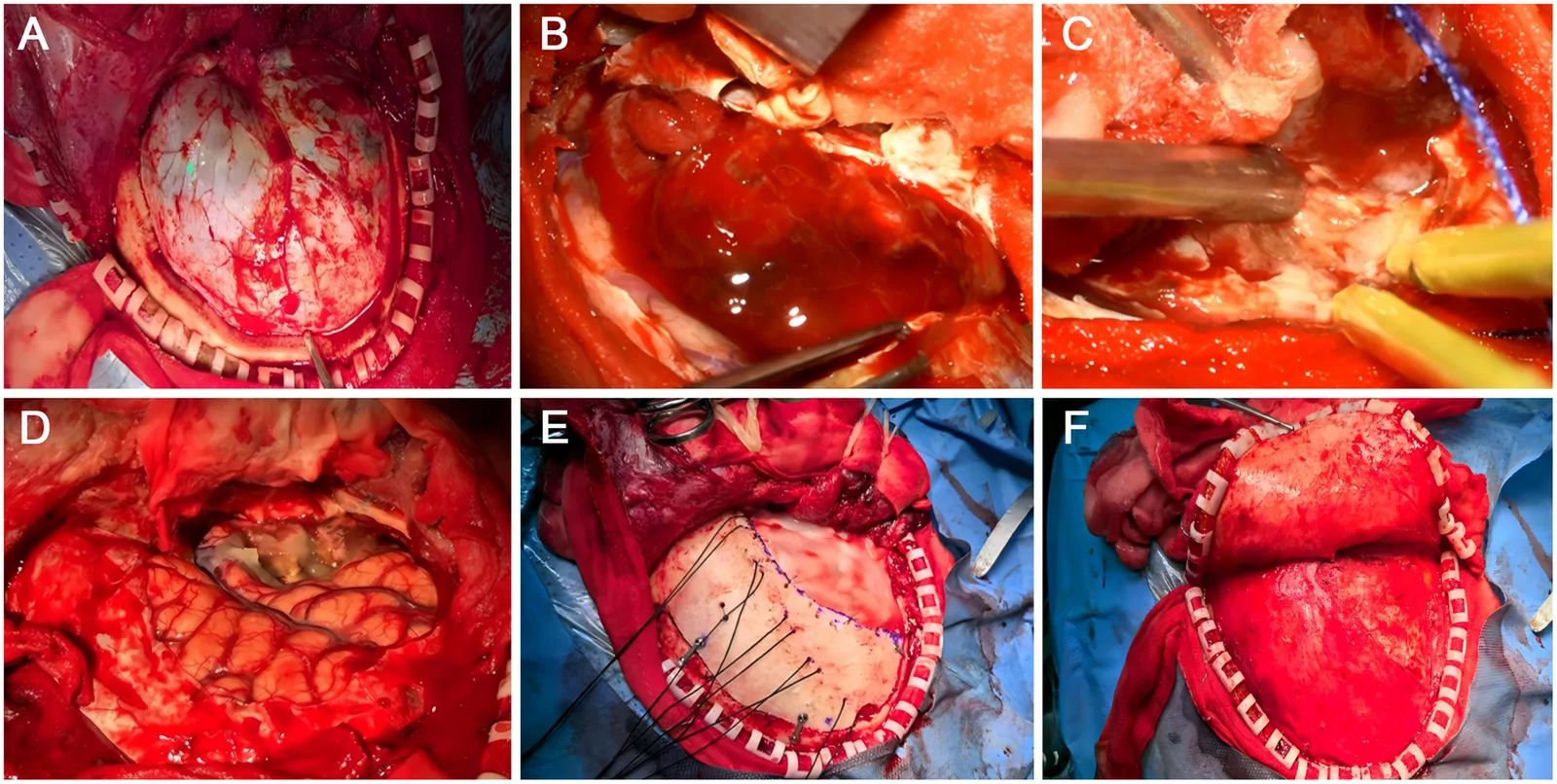
Intraoperative photographs. **(A)** After craniotomy, the tumor was observed to invade the dura mater, with increased dural tension. **(B–D)** Intratumoral necrosis and old hemorrhage were identified. The grayish-red tumor protruded into the brain parenchyma and infiltrated adjacent brain tissue. **(E,F)** Subtemporal decompression was achieved by removing a 6 × 6 cm portion of temporal bone beneath the temporalis muscle.

Histological examination revealed oval to short spindle-shaped tumor cells with high cellularity, brisk mitotic activity, and palisading necrosis. Tumor invasion of the temporal bone and skeletal muscle was identified (Figures 3A–C). Immunohistochemistry showed positivity for glial fibrillary acidic protein (GFAP), oligodendrocyte transcription factor 2 (OLIG2), and S-100, p53 positivity of variable intensity, and a Ki-67 labeling index of approximately 15%. Immunohistochemistry for SOX10 was negative (Supplementary Figure S1). Nuclear expression of ATRX and integrase interactor 1 (INI-1) was retained. Representative immunohistochemical findings are shown in Figures 3D–I. Immunostaining for IDH1, somatostatin receptor 2 (SSTR2), and epithelial membrane antigen (EMA) was negative. BRAF V600E (VE1) immunohistochemistry and OLIG2 immunohistochemistry specifically documenting the skeletal-muscle-infiltrating component could not be performed because suitable additional tissue was unavailable, which represents a limitation of the pathological documentation.

**Figure 3:**
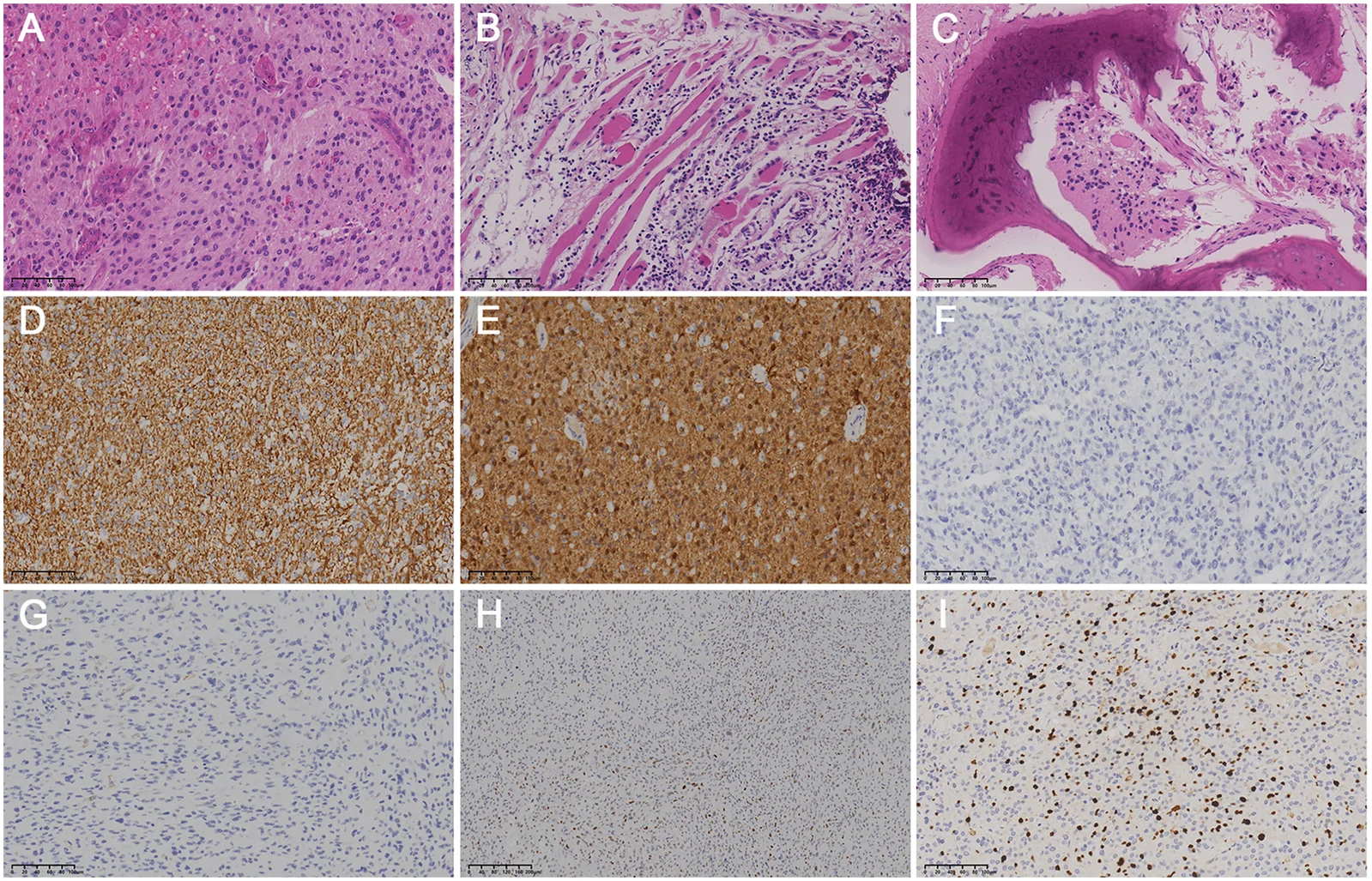
Hematoxylin and eosin (H&E) staining and immunohistochemistry. H&E staining (×20) showed intracranial tumor **(A)**, skeletal muscle infiltration **(B)**, and skull invasion **(C)** Immunohistochemistry (×20) demonstrated diffuse GFAP positivity **(D)**, S-100 positivity **(E)**, IDH1 negativity **(F)**, SSTR2 negativity **(G)**, variable p53 positivity (weak to strong; H), and a Ki-67 labeling index of approximately 15% **(I)**.

Targeted next-generation sequencing was performed using a commercial hybrid-capture 919-gene brain tumor panel (Brain Tumor 919-Gene Panel, tissue assay; Genetron Health, Beijing, China). The estimated tumor-cell fraction was 20%, mean sequencing depth was 1,921.69x, 94.14% of target regions achieved at least 20% of the mean depth, and the Q30 rate was 95.47%. No pathogenic mutation was detected in IDH1 or IDH2, molecularly confirming IDH-wildtype status. The assay identified EGFR amplification, homozygous deletion of CDKN2A/B, TERT promoter C250 T (VAF, 30.2%), and BRAF p.V600E (c.1799T > A; VAF, 28.8%). TP53 was wild-type by sequencing. MGMT promoter methylation was not detected. No chromosome 7 gain/amplification or chromosome 10 loss was detected; however, given the estimated tumor-cell fraction of only 20%, this negative +7/−10 result should be interpreted cautiously. Fluorescence *in situ* hybridization co-assessed 1p and 19q and showed intact 1p with isolated 19q deletion; the exact commercial probe identifiers were unavailable. Because canonical whole-arm 1p/19q codeletion was absent and the tumor was IDH-wildtype, the isolated 19q finding did not support oligodendroglioma and was not considered diagnosis-defining. On the basis of the integrated histopathological, immunohistochemical, and molecular findings, the tumor was diagnosed as glioblastoma, IDH-wildtype, CNS WHO grade 4.

## Discussion

Extradural extension and skull invasion are uncommon patterns of GBM spread beyond the brain parenchyma. When extracranial dissemination is reported, it is usually secondary to neurosurgical intervention, radiotherapy, or other treatment-related disruption of anatomical barriers. In contrast, primary extracranial extension in a patient without prior surgery or adjuvant therapy is exceedingly rare. Previously reported cases are summarized in Table 1.

**Table 1:** Previously reported cases of primary extracranial extension or metastasis of high-grade glioma without prior surgical or adjuvant treatment.

Case study	Age/Sex	Lesion location	Extracranial extension	Imaging findings	Clinical features	Histopathology	Other genetic features	IDH status	MGMT status
Shuangshoti et al. (1987) (11)	69/F	left temporoparietooccipital lobe	Galea aponeurotica, parieto-occipital skull, and dura mater	Inhomogeneous ring-enhancing mass	Headache, stupor, nausea	Glioblastoma multiforme (GFAP-positive)	NA	NA	NA
Shuangshoti et al. (1987) (11)	30/M	right frontotemporal lobe	Dura mater and sphenoid bone	Midline structures shifted 2 cm to the left	Headache, bilateral blurred vision, anorexia	Glioblastoma multiforme (GFAP-positive)	NA	NA	NA
Shuangshoti et al. (1987) (11)	25/F	Right subfrontal and temporal regions	Adjacent dura mater, sphenoid bone, ethmoid bone, and orbit	Destruction of the right ethmoid bone and posterior right orbit	Seizure, diplopia, right proptosis	Glioblastoma multiforme (GFAP-positive)	NA	NA	NA
Rainov et al. (1996) (10)	66/F	left temporal lobe	Paranasal sinuses, orbit, temporal bone, and pterygoid muscles	Slight perifocal edema of surrounding white matter	Headache, left-sided facial pain, painful temporal swelling	Glioblastoma multiforme (strong GFAP- positive)	Functional p53 inactivation; no EGFR or MDM2 overexpression	NA	NA
Sade et al. (2006) (25)	55/M	right temporal lobe	Infratemporal fossa and lateral sphenoid sinus	Right temporal mass involving the cavernous sinus and infratemporal fossa and extending into the sphenoid sinus	Progressive headache	Gliosarcoma (GFAP- and S-100-positive)	No EGFR amplification or 1p/19q loss by FISH	NA	NA
Thakar et al. (2015) (15)	53/M	right temporal lobe	Right sphenoid, maxillary sinus, squamous temporal bone, and orbit	Right temporal and subtemporal lesion with invasion into the infratemporal fossa	Right-sided proptosis and right temporal swelling	Glioblastoma multiforme (GFAP-positive)	NA	NA	NA
Nguyen et al. (2016) (26)	44/F	right temporal lobe	Orbit, maxillary sinus, right pterygoid, masseter, and temporalis muscles	Increased midline shift, mass effect, and vasogenic edema	Difficulty opening the right jaw, cheek and temporal swelling, severe headache with retroauricular pain radiating to the neck	Gliosarcoma (GFAP-positive)	ATRX expression retained	IDH-wildtype (IDH1 R132H-negative by IHC; no IDH1 or IDH2 mutation detected by pyrosequencing)	NA
Taghipour Zahir et al. (2017) (27)	60/M	Right frontal lobe and corpus callosum	Right frontal bone with lytic areas	Dura and galea infiltrated; large areas of necrosis and hemorrhage	Seizures and egg-sized right frontal parasagittal mass	Glioblastoma multiforme (GFAP-, S-100-, CD68-, OLIG2-, and p53-positive)	NA	NA	NA
Stib et al. (2017) (28)	42/F	Right frontal lobe	Anterior skull base and intranasal extension	Mass extended through the anterior skull base into the right ethmoid and maxillary sinuses and nasal cavity	Chronic sinusitis, rhinorrhea, and near-total obstruction of the right nasal passage	Anaplastic oligoastrocytoma (GFAP-, p53-, and IDH1-positive)	No deletion of either 1p or 19q by FISH	IDH1-positive by IHC	NA
Present case	37/F	Right temporal lobe	Dura, temporal bone, and temporalis muscle	Hemorrhagic hypervascular temporal mass with contiguous transdural and calvarial extension	Headache and vomiting	Glioblastoma, IDH-wildtype, CNS WHO grade 4 (GFAP- and OLIG2-positive)	BRAF V600E; TERT C250 T; EGFR amplification; CDKN2A/B homozygous deletion; isolated 19q deletion	IDH1/IDH2 wild-type by NGS	Unmethylated

F, female; M, male; GFAP, glial fibrillary acidic protein; IDH, isocitrate dehydrogenase; IHC, immunohistochemistry; FISH, fluorescence *in situ* hybridization; MGMT, O6-methylguanine-DNA methyltransferase; NA, not available; NGS, next-generation sequencing; OLIG2, oligodendrocyte transcription factor 2.

The dura mater and calvarium are generally considered important barriers to glioma spread (12). Extracranial extension may be recognized more often in younger or otherwise healthier patients, partly because longer survival provides more time for unusual dissemination patterns to become clinically evident (13). Nevertheless, spontaneous transdural and calvarial invasion remains rare even in this context.

Several mechanisms have been proposed to explain extracranial extension of GBM, including spread through perivascular or dural slits, direct dural destruction, extension along cranial nerves, and lymphatic dissemination (14, 15). Treatment-related dural defects after surgery or radiotherapy may facilitate tumor transgression in many cases (16). Other reports have suggested that severe intracranial hypertension can promote extracranial diffusion distant from an operative site (17). Rare untreated malignant astrocytomas have been described with spontaneous invasion of the superior sagittal sinus and systemic metastasis to bone marrow and lymph nodes (16). In temporal-base GBM, chronic elevation of intracranial pressure has been proposed to drive tumor penetration through perivascular slits (18). Direct orbital extension has also been attributed to invasion along white-matter tracts (10). In addition, extracranial spread to the lung, pleura, mesentery, liver, and soft tissue supports the possibility of hematogenous or lymphatic routes, and renewed evidence for intracranial lymphatic drainage has strengthened interest in lymphatic pathways (13, 19–21).

In the present case, imaging and operative findings showed contiguous involvement of the dura, temporal calvarium, and temporalis muscle in the absence of previous surgery, chemotherapy, or radiotherapy. There was no clear evidence of cranial-nerve involvement, marked intracranial hypertension, infratemporal fossa invasion, or a primary extra-axial malignant lesion. These observations are most compatible with direct dural destruction and contiguous extension into adjacent bone and soft tissue. However, a route of spread cannot be established from a single case, and alternative pathways or tumor-specific biological factors cannot be excluded. Accordingly, the term ’spontaneous’ is used descriptively to indicate occurrence before iatrogenic disruption of anatomical barriers, rather than to establish a unique biological mechanism. GBM with extracranial extension may also be radiologically and clinically misinterpreted as a primary skull-base, meningeal, or soft-tissue tumor, particularly when the extracranial component is prominent.

The molecular profile of this tumor is relevant to the integrated diagnosis and differential diagnosis. Under the current WHO framework, an adult diffuse astrocytic glioma with IDH-wildtype status and molecular features such as EGFR amplification or TERT promoter mutation supports the diagnosis of glioblastoma, IDH-wildtype, CNS WHO grade 4 (1). In this case, IDH1/IDH2 were wild-type by sequencing, and EGFR amplification, TERT promoter mutation, and CDKN2A/B homozygous deletion were identified despite the absence of the +7/−10 signature. The isolated 19q deletion, together with intact 1p and IDH-wildtype status, did not represent canonical 1p/19q codeletion and was therefore not considered diagnostically supportive of oligodendroglioma.

BRAF V600E deserves particular consideration because it is enriched in glioblastomas with epithelioid morphology and is also frequently detected in pleomorphic xanthoastrocytoma (PXA). The combination of BRAF V600E and CDKN2A/B homozygous deletion is therefore not specific to glioblastoma. In the present case, however, the sampled sections showed an infiltrative high-grade glial neoplasm composed predominantly of oval-to-spindle pleomorphic cells, with brisk mitotic activity and palisading necrosis. Neither a predominant population of large epithelioid cells nor a xanthomatous or otherwise classic PXA-like component was identified. In this morphological context, IDH1/IDH2 wild-type status, EGFR amplification, and a TERT promoter mutation further supported the diagnosis of glioblastoma, IDH-wildtype, CNS WHO grade 4. We therefore favored conventional glioblastoma over PXA or glioblastoma with predominant epithelioid morphology (22, 23). Genome-wide DNA methylation profiling was technically feasible but was not pursued because the patient declined additional molecular testing after its potential diagnostic value had been explained. From a therapeutic perspective, the BRAF V600E alteration remains potentially actionable because combined BRAF/MEK inhibition has shown activity in recurrent or refractory BRAF V600E-mutant high-grade gliomas (24).

The differential diagnosis also included gliosarcoma, metastatic melanoma, high-grade meningioma, and other primary dural or skull-base malignancies. Gliosarcoma was not favored because a biphasic glial and sarcomatous architecture was not identified. Metastatic melanoma was considered because of the pigmented cutaneous lesions, S-100 expression, and BRAF V600E mutation. However, the intracerebral origin, GFAP and OLIG2 positivity, infiltrative glial morphology, palisading necrosis, EGFR amplification, and TERT promoter mutation favored a primary glial neoplasm. SOX10 immunohistochemistry was negative, providing additional evidence against melanocytic differentiation. Preoperative PET/CT did not identify another metabolically active extracranial lesion or primary malignancy, although a negative PET/CT examination cannot exclude melanoma. Moreover, the cutaneous lesions were not evaluated dermatologically or histopathologically, and immunohistochemistry for HMB-45 and Melan-A was not performed. Therefore, metastatic melanoma was considered less likely but could not be excluded with absolute certainty, which remains a limitation of the present report. Negative immunostaining for SSTR2 and EMA, together with glial marker expression and an infiltrative intra-axial component, argued against meningioma. Overall, the combined morphological, immunophenotypic, radiological, and molecular findings favored glioblastoma, IDH-wildtype.

## Conclusion

In conclusion, we report a rare case of glioblastoma, IDH-wildtype, CNS WHO grade 4, with transdural, temporal-calvarial, and temporalis-muscle invasion identified before any surgical or chemoradiotherapeutic intervention. This case emphasizes that GBM should remain in the differential diagnosis of destructive temporal or skull-base masses with intracranial and extracranial components. The pattern is compatible with direct contiguous extension, but the route cannot be proven from a single case. Careful integration of imaging, histopathology, immunohistochemistry, and molecular testing is essential for accurate diagnosis and appropriate postoperative management.

## Data Availability

The original contributions presented in the study are included in the article/Supplementary Material, further inquiries can be directed to the corresponding authors.
